# Two consecutive large outbreaks of *Salmonella* Muenchen linked to pig farming in Germany, 2013 to 2014: Is something missing in our regulatory framework?

**DOI:** 10.2807/1560-7917.ES.2017.22.18.30528

**Published:** 2017-05-04

**Authors:** Anika Schielke, Wolfgang Rabsch, Rita Prager, Sandra Simon, Angelika Fruth, Rüdiger Helling, Martin Schnabel, Claudia Siffczyk, Sina Wieczorek, Sabine Schroeder, Beate Ahrens, Hanna Oppermann, Stefan Pfeiffer, Sophie Susann Merbecks, Bettina Rosner, Christina Frank, Armin A. Weiser, Petra Luber, Andreas Gilsdorf, Klaus Stark, Dirk Werber

**Affiliations:** 1 Robert Koch Institute (RKI), Berlin and Wernigerode, Germany; 2 Postgraduate Training for Applied Epidemiology (PAE, German Field Epidemiology Training Programme), Robert Koch Institute, Berlin, Germany; 3 European Programme for Intervention Epidemiology Training (EPIET), European Centre for Disease Prevention and Control (ECDC), Stockholm, Sweden; 4 Saxon State Ministry of Social Affairs and Consumer Protection, Dresden, Germany; 5 Brandenburg State Office of Occupational Safety, Consumer Protection and Health, Potsdam, Germany; 6 Thuringian State Authority for Consumer Protection, Bad Langensalza, Germany; 7 Agency for Consumer Protection of the Federal State of Saxony-Anhalt, Magdeburg, Germany; 8 Ministry of Labour and Social Affairs Saxony-Anhalt, Magdeburg, Germany; 9 State Health Authority Saxony, Chemnitz, Germany; 10 The Federal Institute for Risk Assessment, Berlin, Germany; 11 Federal Office of Consumer Protection and Food Safety, Berlin, Germany; 12 State Office for Health and Social Affairs, Berlin, Germany

**Keywords:** food-borne infections, outbreaks, *Salmonella*, pork, pig, tracing

## Abstract

In 2013, raw pork was the suspected vehicle of a large outbreak (n = 203 cases) of *Salmonella* Muenchen in the German federal state of Saxony. In 2014, we investigated an outbreak (n = 247 cases) caused by the same serovar affecting Saxony and three further federal states in the eastern part of Germany. Evidence from epidemiological, microbiological and trace-back investigations strongly implicated different raw pork products as outbreak vehicles. Trace-back analysis of *S*. Muenchen-contaminated raw pork sausages narrowed the possible source down to 54 pig farms, and *S*. Muenchen was detected in three of them, which traded animals with each other. One of these farms had already been the suspected source of the 2013 outbreak. *S*. Muenchen isolates from stool of patients in 2013 and 2014 as well as from food and environmental surface swabs of the three pig farms shared indistinguishable pulsed-field gel electrophoresis patterns. Our results indicate a common source of both outbreaks in the primary production of pigs. Current European regulations do not make provisions for *Salmonella* control measures on pig farms that have been involved in human disease outbreaks. In order to prevent future outbreaks, legislators should consider tightening regulations for *Salmonella* control in causative primary production settings.

## Introduction

Salmonellosis is a zoonotic enteric disease caused by a multitude of non-typhoidal serological variants of *Salmonella enterica*.

The number of human cases of salmonellosis in Europe reported to the European Centre for Disease Prevention and Control (ECDC) has been declining remarkably since the first report in 1995 [1,2]. This is mainly attributable to a reduction in disease cases caused by *S. enterica* subsp. *enterica* serovar Enteritidis (*S*. Enteritidis), the most prevalent serovar in Europe. Human *S*. Enteritidis infections are primarily attributed to poultry and eggs [3]. Measures regarding hygiene and immunisation of chicks and young hens in broiler chicken production and in laying hens are held responsible for the decline of human cases [2,4].

With the diminishing importance of poultry as source of human salmonellosis in Europe, the relative importance of pig-related salmonellosis has increased. *S*. Typhimurium is the second most frequent serovar isolated from salmonellosis cases in Europe [2] and the most prevalent serovar identified in European pigs [4]. The number of human cases reported to the ECDC caused by this serovar has decreased only slightly since 2008. Furthermore, detection of the monophasic variant of *S*. Typhimurium remarkably increased since it was first reported to ECDC in 2010 [2]. Human cases with *S*. Typhimurium and its monophasic variants are primarily related to swine [5].

**Figure 1:**
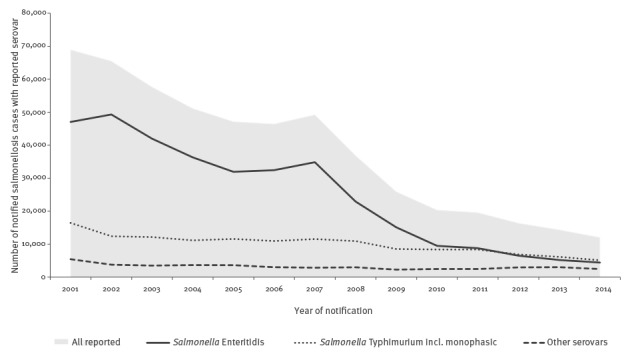
Number of notified salmonellosis cases with reported serotype in Germany, 2001–2014

In Germany, salmonellosis was the most frequently reported bacterial disease until 2005 [6]. Very similar to the situation in Europe, there has been a decreasing trend in salmonellosis in Germany since 1992; mainly due to a reduced incidence of *S*. Enteritidis [7]. However, the number of cases caused by *S*. Typhimurium and by other *Salmonella* serovars has been relatively constant between 2001 and 2014 (Figure 1) [6]. Large salmonellosis outbreaks investigated in Germany in recent years were caused by *S*. Typhimurium or rare serovars. The majority of these outbreaks have been attributed to the consumption of raw pork and products thereof [8-10].

Incl. monophasic: including monophasic variant.

In June 2013, public health authorities in the federal state of Saxony, eastern Germany, investigated a salmonellosis outbreak caused by the rare serovar *S*. Muenchen, which antigenically is a group C2-C3 *Salmonella*. In that state, an annual median of three cases (range: 1–6 cases) had been reported from 2005 to 2012. During the outbreak, between 25 June and 7 August, a total of 203 cases were reported. The median age was 50 years (interquartile range (IQR): 39–62 years) and 56% of cases (113/203) were male. A convenience subset of strains (n = 21) was sent to the National Reference Centre for *Salmonella* (NRC) in Wernigerode where pulsed-field gel electrophoresis (PFGE) analysis suggested that cases were epidemiologically linked (data not shown). Raw pork was the suspected vehicle based on positive tested food specimens. Based on trace-back analysis and the detection of *S.* Muenchen on a pig breeding farm in a routine specimen in temporal relation to the outbreak, this farm was considered as the likely source of the outbreak (data not shown). Measures to stop the outbreak were mainly applied at the level of meat processing addressing severe hygienic deficits identified there.

Almost one year later in June 2014, the public health authority in Brandenburg, a federal state bordering Saxony, informed the Robert Koch Institute (RKI) of an increase in reported *S*. Muenchen infections. At that time, increased case numbers of *S*. Muenchen were also reported from Saxony, Saxony-Anhalt and Thuringia, all states located in eastern Germany. Coincident to the increase of *S*. Muenchen cases, routine or targeted testing detected *S*. Muenchen and/or *Salmonella* type C in different pork products. A possible resurgence of the salmonellosis outbreak that had occurred in Saxony the year before was hypothesised.

We conducted a multistate inter-sectoral outbreak investigation in 2014 to strengthen or refute the evidence for raw pork or products thereof as vehicles of infection and to identify the source in order to stop the outbreak. A possible connection to the outbreak in 2013 and potential reasons for resurgence were also investigated. The existing legal basis was reviewed to identify possible gaps in the regulatory frameworks that are intended to safeguard consumers against *Salmonella* along the pork food production chain.

## Methods

### Outbreak case definition

For surveillance in Germany, the national case definition for salmonellosis includes patients presenting with the typical clinical picture of an acute salmonellosis (with at least one of the following symptoms: abdominal pain, diarrhoea, vomiting or fever (>38.5°C)) and either isolation of *Salmonella* spp. (laboratory confirmation) or an epidemiological link to a laboratory-confirmed case, as well as laboratory-confirmed *Salmonella* infections with an unknown or untypical clinical picture.

We defined outbreak cases as persons notified with *S.* Muenchen or *Salmonella* of group C/C2-C3 infections in affected federal states (Brandenburg, Saxony, Saxony-Anhalt, Thuringia) who fulfilled the national surveillance case definition with symptom onset between 26 May 2014 and 03 August 2014 or – if onset dates were missing – with notification weeks 22 to 31 (26 May to 03 August) of the year 2014.

### Epidemiological investigations

Case information was analysed daily regarding time, place and person (age, sex, laboratory results and deaths). Information was shared by regular reports and telephone conferences, between the members of the inter-sectoral multistate outbreak team, which involved authorities for human health and food safety.

Staff of local health authorities attempted to interview all adult notified cases (18 years and older) with reported salmonellosis starting from 26 May 2014 using a specifically designed questionnaire. The questionnaire asked about consumption of pork and pork products as well as points of both purchase and consumption of the products, e.g. butcher shops and restaurants. The aim of the interviews, which took place until 14 July 2014, was to identify common points of purchase (at least 2 mentions) to provide possible starting points for trace-back investigations along the food production chain.

### Cohort study among staff of a nursing home

We conducted a cohort study among staff of a nursing home with cases among staff and residents, in which raw pork sausages from unopened packages had tested positive for *S*. Muenchen. Participants were asked via an online questionnaire about symptoms, the meal they had participated in (e.g. breakfast or lunch) and the food they had consumed in the canteen of the nursing home in May and June 2014. For this study, cases were defined as staff reporting diarrhoea with symptom onset between 29 May 2014 and 09 June 2014 or detection of *S*. Muenchen or untyped *Salmonella* in a stool specimen taken in May or June 2014. Risk ratios (RR) and corresponding 95% confidence intervals (CI) were calculated.

### Investigations by food safety authorities

In order to identify the food vehicle and the source of contamination, food safety authorities conducted risk-based inspections in kitchens of institutional caterers, butcher shops and supermarkets that were possibly involved in the outbreak. During these inspections, specimens of different food items with a focus on pork products and environmental swab samples were taken and analysed. Food specimens that tested positive for the outbreak strain were traced back along the food production chain to their origin (e.g. food business operator producing raw pork sausages) and further to the level of primary production. Food business operators and slaughterhouses identified through trace-back investigations were inspected and sampled. Pig farms identified by trace-back analysis and located in Brandenburg and Saxony were visited to take surface swabs and to collect animal faeces for further testing. 

All information generated by the authorities of the federal states on sampling, testing, inspections, and trace-back of positive foods was summarised in situation reports by the Federal Office of Consumer Protection and Food Safety (BVL). Additionally, the collected supply chain information was provided to the central authorities for import into ‘FoodChain-Lab’, a relational database with integrated consistency and plausibility checks developed at the Federal Institute for Risk Assessment (BfR) [11,12]. This tool was used by the BfR for analysis and visualisation of the investigated supply chains.

### Microbiological investigations

Primary diagnostic laboratories in the affected region were asked to fully serotype all detected *Salmonella* strains from patients and to forward *S*. Muenchen isolates as well as *Salmonella* isolates of group C or C2-C3 to the NRC for *Salmonella* for subtyping using PFGE according to the Pulse-Net protocol [13]. Isolates from different sources (human, food and environment) were compared with each other and to isolates from the salmonellosis outbreak in Saxony in 2013.

### Review of legal framework

Finally, we reviewed the existing legal provisions targeting consumer protection against *Salmonella* when eating raw pork products to identify possible gaps in the regulatory framework.

## Results

### Descriptive epidemiological investigations

**Figure 2:**
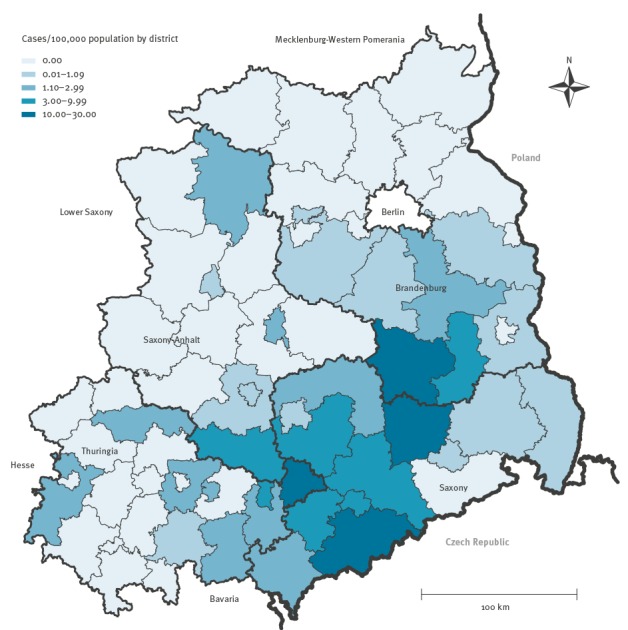
Reported incidence of salmonellosis per district (number of outbreak cases/100,000 population) in four federal states affected by an outbreak, eastern Germany, May–August 2014 (n = 247 cases)

**Figure 3:**
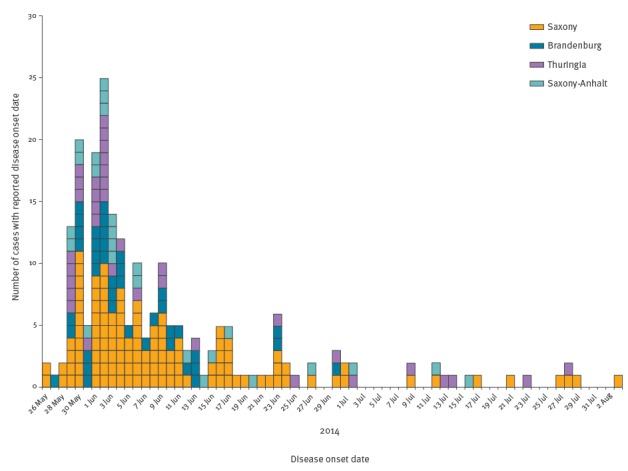
Reported cases with *Salmonella* Muenchen and known date of symptom onset, Germany, 26 May–3 August 2014 (n = 217 cases)

In total, 247 notified patients with salmonellosis met the outbreak case definition. Most of them were laboratory-confirmed (n = 237) and of these, 90% (n = 213) were serotyped as *S*. Muenchen, the remaining were typed only to the group level. Most outbreak cases were reported from Saxony (n = 139; 56%). Likewise, districts in or bordering Saxony reported the highest incidence during the outbreak period between 26 May 2014 and 03 August 2014 (11–28 cases/100,000 population) (Figure 2). Median age of the outbreak cases was 56 years (IQR: 42–71 years); 54% were male (n = 133). Of all outbreak cases, 12% (n = 30) were hospitalised after their symptom onset; four patients died (all female, age range 81–93 years). For one of these patients, salmonellosis was reported as cause of death; for the other three patients, deaths were attributed to causes other than salmonellosis but without any further information. For outbreak cases with a known symptom onset (n = 217), most contracted disease between 29 May 2014 and 23 June 2014 (n = 185; 85%) (Figure 3). At least three nursing homes were affected by the outbreak (two in Saxony and one nursing home in Brandenburg).

National, federal state and district borders are distinguished on the graph by line thickness, with the thickest lines for national borders, followed by federal state borders and district borders.

### Cohort study among staff of a nursing home

Staff of local health authorities interviewed 160 patients with reported salmonellosis. Of these, four did not belong to the outbreak and eight did not give their consent or could not be reached, resulting in 148 questionnaires that could be analysed for consumption of pork, pork products and points of purchase or localities of consumption of the products. Of these 148 patients, 80% (n = 119) reported the consumption of pork and products thereof in the three days before symptom onset, and of these, 85% (n = 101) raw pork consumption. In total, 11 common points of purchase were identified (mainly supermarkets and butcher shops) and the information was forwarded to food safety authorities.

### Analytical epidemiological investigations

In the cohort study, 27 of 64 staff members of a nursing home affected by the outbreak completed the online questionnaire (response rate: 42%). Of these, six were defined as cases. Median age of participants was 48 years (IQR: 28–64 years) and the majority was female (n = 23 participants). Staff members eating their breakfast in the canteen at work during the time of the outbreak were 10-times more likely to become a case than staff members eating their breakfast elsewhere (RR: 10; 95% CI: 1.4–73; 5 cases among 9 exposed persons (attack rate: 56%) and one case among 18 non-exposed persons (attack rate 6%)). During breakfast, several types of raw pork sausages were served in the canteen during the respective time period.

### Investigations by food safety authorities

The hypothesis that raw pork products represented the vehicle of this outbreak was generated at an early stage of the investigation, since *S*. Muenchen was detected in two samples taken for routine testing of food to determine microbiological parameters. These were different pork products like minced pork intended for raw consumption, and a raw pork sausage called ‘Knacker’, also meant to be consumed raw. Additionally, a specimen of brine used for meat preparations tested positive. 

During the outbreak investigation, food safety authorities collected further 117 food specimens. Two raw pork sausages, also intended for raw consumption, from unopened packages collected from the kitchen of the affected nursing home where we conducted the cohort study, tested positive for *S*. Muenchen as well (‘Braunschweiger’ and ‘Schinken-Teewurst’). 

In total, as a result of the routine testing and outbreak investigations, four of 119 food specimens and one specimen of brine tested positive for *S*. Muenchen. In contrast, none of 227 surface swabs taken in kitchens of nursing homes, butcher shops and slaughterhouses tested positive.

Food safety authorities conducted a trace-back investigation starting with the two raw pork sausages from the nursing home that had tested positive for *S*. Muenchen and the minced pork and ‘Knacker’, which had been sampled during routine investigations and had tested positive for *S*. Muenchen as well. The brine was not further traced back because of probable cross-contamination. The trace-back investigation identified different meat processors and slaughterhouses, which were all visited, sampled and investigated for the presence of *Salmonella* – all tested negative. The slaughterhouses were supplied by 54 pig farms. Of these, 23 pig farms (animal faeces and surfaces in the environment of the animals, e.g. the floor of the pig houses) were tested using boot swabs. Swabs from three of the farms located in Saxony tested positive for *S*. Muenchen and isolates were sent to the NRC (n = 8). One of these farms had already been the suspected source of the 2013 outbreak. The three farms belonged to the same owner and were specialised in pig breeding, rearing and fattening, respectively. Pigs were traded among the three farms. The fattening pig farm was subject to the mandatory German *Salmonella* control programme that involves testing pigs for *Salmonella*-specific antibodies pre-slaughter and had been grouped into category I representing a low prevalence. 

### Microbiological investigations of human and environmental samples

*S*. Muenchen was confirmed at the NRC for 143 human isolates from the 2014 outbreak, 52 of which were subtyped. Of those, 47 isolates showed an identical outbreak PFGE pattern (ECDC nomenclature XbaI.1056); four isolates had slightly different band patterns and were also classified as outbreak-related. One isolate was classified as not related to the outbreak. 

Furthermore, the outbreak PFGE pattern was detected in the eight isolates derived from the three pig farms, three pork-based food specimens (i.e. the Knacker found during routine testing and the two raw pork sausages found during the outbreak investigation in the nursing home) and the brine. One of the food specimens (minced pork, which was found with *S*. Muenchen during routine testing) showed the same pattern variation as the outbreak-related human strains mentioned above. Remarkably, the outbreak PFGE pattern had already been identified for human and minced pork isolates during the 2013 outbreak. The NRC-PFGE-database of *S.* Muenchen contains 59 PFGE patterns derived from altogether 218 isolates from humans, food, and animals between 2000 and 2014. The outbreak pattern had been detected before 2013 but only in single isolates from sporadic cases.

### Review of legal framework

**Figure 4:**
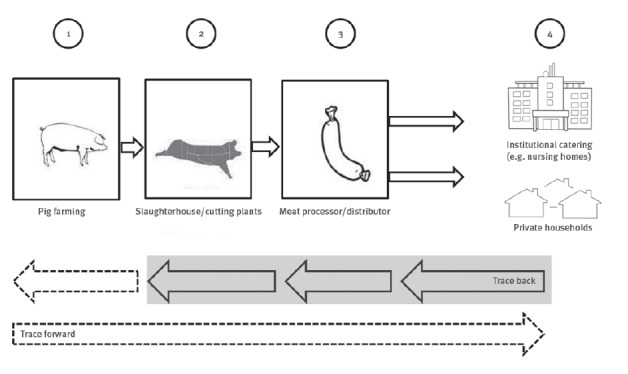
Schematic overview of the pork food production chain ‘from farm to fork’

We identified European Union (EU) regulations, as well as German national laws and recommendations aiming to form a multi-barrier to protect consumers from *Salmonell*a with provisions at the different stages of the pork production chain, from farm to fork (Figure 4).

1 Primary production; 2 Meat productions including slaughterhouses and meat cutting plants (where the carcasses are cut into smaller pieces); 3 Meat processing; 4 Consumers.

The greyish background highlights the stages until where trace-back investigations are usually conducted.

Sources of the images: Fotolia #67699729; Rainer Zenz – own work, https://commons.wikimedia.org/w/index.php?curid=23986913; http://publicdomainvectors.org/de/kostenlose-vektorgrafiken/Vektor-Illustration-fleckig-Wurst/31268.html; http://publicdomainvectors.org/de/kostenlose-vektorgrafiken/Krankenhaus-Geb%C3%A4ude-Linie-Kunst-Vektor-Grafiken/24525.html

#### Stage 1 (primary production)

Regulation (EC) No 2160/2003 provides the general framework for the control of food-borne zoonotic agents in the EU demanding the establishment of targets for the reduction of prevalence of specified zoonoses and zoonotic agents (e.g. for all *Salmonella* serotypes with public health significance in breeding herds of pigs) [14]. In Germany two regulations are implemented regarding *Salmonella* in pigs at primary production: the pig production hygiene regulation (‘Schweinehaltungshygieneverordnung’, SchHaltHygV) [15] and the pig *Salmonella* ordinance (‘Schweine-Salmonellen-Verordnung’, SchwSalmoV) [16]. The former generally describes hygienic requirements for pig farms, specifying that perished or certain sick animals have to be tested for the causative agent and epidemiological investigations have to be conducted to identify the cause. The pig *Salmonella* ordinance mandates the routine testing of fattening pigs for the presence of *Salmonella*-specific antibodies. Pig farms are categorised based on the resulting seroprevalence into three categories with category I denoting pig farms with the lowest seroprevalence (0–20%). Pig farms of category II show seroprevalences between 21% and 40%. Measures to reduce seroprevalence on the farm are only obligatory for pig farms belonging to category III with a seroprevalence of more than 40%.

Regulation (EC) No 178/2002 [17] lays down general requirements for food safety in Europe, but basically does not apply to pig farms because it does not define food producing animals as food. The German national law concerning The Foods, Consumer Goods and Feedstuffs Code (‘Lebensmittel-Bedarfsgegenstände- und Futtermittelgesetzbuch’, LFGB) [18] aligns with that definition.

#### Stage 2 (slaughterhouse)

At the stage of the slaughterhouse, process hygiene criteria stated in Regulation (EC) No 2073/2005 [19] apply. This regulation specifies that when more than 6% of a minimum of 50 tested swine carcasses (3/50) are found positive for *Salmonella* using cultural techniques, improvements in slaughter hygiene have to be taken, and process controls, the origin of animals, and biosecurity measures on the farms of origin have to be reviewed.

#### Stage 3 (meat processor/distributer)

Minced meat, meat preparations and meat products intended to be eaten raw (food safety criteria, also Regulation (EC) No 2073/2005), are not allowed to contain *Salmonella* [19]. Five pooled specimens of at least 25g each taken on one day per week have to test negative using cultural techniques.

#### Stage 4 (consumer)

To protect especially vulnerable population groups, the BfR recommends that raw pork and products thereof should not be served in institutional catering (e.g. nursing homes). This recommendation was published in 2011 [20]. Next to that, the German Institute for Standardisation (DIN) published the DIN standard 10506:2012–3 on ‘Food hygiene – Mass catering’, which gives detailed advice to caterers providing food for vulnerable persons not to serve raw foods of animal origin such as raw minced meat and raw pork sausages [21].

## Discussion

Our epidemiological, microbiological and product tracing investigations suggest that (i) raw pork products were the vehicles in the outbreak of *S*. Muenchen infections in 2014, (ii) the outbreaks in 2013 and 2014 were connected and (iii) the common source was at the farm-level. The negative tested surface swabs taken at slaughterhouses, meat processors and butcher shops in 2014 are compatible with good hygiene practices at the stages following primary production.

Raw pork and products thereof are a traditional and popular food in Germany, particularly in the northern and eastern parts [22]. Consumption of raw pork is also the cardinal risk factor for yersiniosis [22] and likely also important for toxoplasmosis in Germany [23]. Dependent on the region in Germany, different raw pork products are consumed, including, for example, spreadable sausages. These may sometimes not even be recognised by the consumer as containing raw pork, and hence risk awareness regarding consumption of these products is likely low.

Despite national recommendations in Germany to the contrary [20,21], raw pork products were served in at least three affected nursing homes. In one of these homes, the reported reason was that many elderly people strongly requested this traditional food, e.g. spreadable sausages containing raw pork. We also suspected that even when raw pork products were not served in a nursing home, visitors would provide residents with these products. For the other identified institutions, it was unclear whether failure to follow this recommendation was due to deliberate non-compliance or lacking knowledge.

The resource-intensive trace-back investigations, involving food safety authorities from all levels, were pivotal in identifying the three pig farms as likely source of the outbreak in 2014. Despite this and other successes in the recent past [12], product tracing (back and especially forward) investigations are seldom conducted in Germany and Europe. This may be one reason why the source of many food-borne outbreaks is not identified. We advocate conducting product tracing investigations more frequently, especially in the investigation of geographically diffuse food-borne outbreaks. As this outbreak exemplifies, without identifying the source, the risk of further illnesses or outbreaks including deaths may remain. *Salmonella*, often introduced by feed or animal trade [24], may persist on a farm, either in the environment, for example, on surfaces where bacteria may be protected against disinfection in a biofilm matrix, or in the pigs themselves. Intermittent shedding induced by external factors (e.g. change of feed, stress of the animals) or unintentional sloughing of biofilms may then lead to a re-contamination on the farm [25].

To our knowledge, all relevant regulations and laws were followed in the aftermath of the outbreak in 2013. Yet, only one year later the same strain caused another even larger outbreak, including at least one death, showing that the multi-barrier approach to prevent human *Salmonella* infections failed.

When reviewing current provisions to control *Salmonella* in pigs, we identified weaknesses from an infection control point of view. Most notably, competent authorities, such as veterinary control services, have no legal mandate to initiate measures at farms even if they can be linked to human cases. Yet, they should be able to demand mandatory preventive measures at primary production after a food-borne outbreak.

In poultry, measures need to be taken when presence of certain *Salmonella* serovars is suspected. In contrast, presence of *Salmonella*, either antibodies or bacteria, in (asymptomatic) pigs does not require action, at least up to a farm seroprevalence of 40%. The fattening pig farm identified as possible source of the outbreaks had a seroprevalence of less than 20% and was thus grouped into the lowest *Salmonella* prevalence category for pig farms. This case in point adds to the view that antibody prevalence alone inadequately reflects the infection risk posed by pigs [26,27]. Furthermore, the proportion of farms in Germany with low *Salmonella* seroprevalence remained relatively constant or even slightly decreased over time (79% in 2006; 74% in 2016) (personal communication: Sabrina Heß, Qualität und Sicherheit GmbH, Bonn, April 2016) reflecting that the German *Salmonella* control programme failed to notably reduce *Salmonella* prevalence at the farm level. By testing sick or dead animals, as requested by the national legal provisions on pig farm hygiene, *Salmonella* positive pigs may not be detected because human pathogenic *Salmonella* strains mainly cause mild or even asymptomatic infections in pigs [28,29]. Admittedly, control of *Salmonella* in pigs is intricate and eradication of *Salmonella* on pig farms might not be achievable. However, this underscores the need not only for quality control programmes but also for mandatory regulations when farms are linked or suspected to be linked to human salmonellosis cases.

### Limitations

Suspicion of raw pork(-products) as vehicles existed early on in this outbreak due to detection of *S*. Muenchen in several pork specimens. Epidemiological investigations served mainly to support the evidence and did not explore other potential vehicles with the same rigour. The validity of the cohort study is somewhat compromised by the rather low participation and identified only a plausible surrogate (breakfast) as a risk factor – not raw pork products themselves. Yet, evidence from different lines of investigations is consistent and points to raw pork products as outbreak vehicles.

Our review of the regulatory framework focused on Germany. Some requirements anchored in EU regulations remain general, allowing for interpretation. Thus, their implementation into national regulations may not be uniform across Europe.

### Lessons learnt and recommendations for the future

Raw pork remains a risky, yet frequently consumed food product in Germany that may cause salmonellosis and other infectious diseases, e.g. yersiniosis. Recommendations, e.g. not to serve raw pork(-products) to vulnerable populations, are necessary building blocks of food-borne illness prevention strategies, but are apparently not sufficient to prevent salmonellosis outbreaks caused by pork in Germany. We recommend a survey regarding knowledge, attitude and practices or an anthropological approach to understand underlying reasons for non-compliance with guidance documents, which might also improve formulation of future recommendations. Only if missing knowledge about existence of the recommendation was the main reason for non-compliance, intensifying tailored communication would be a sensible strategy. Furthermore, legislators should review the existing regulatory framework regarding protection of the consumer against *Salmonella* in raw pork products with a focus on primary production to critically assess where regulations can be tightened to better prevent future outbreaks and to protect the consumer from infectious diseases. Most notably, we recommend that competent authorities, such as veterinary control services, should have a legal mandate to initiate measures at farms if they can be linked to human cases.
